# Growth of the protozoan parasite *Entamoeba histolytica *in 5-azacytidine has limited effects on parasite gene expression

**DOI:** 10.1186/1471-2164-8-7

**Published:** 2007-01-05

**Authors:** Ibne Karim M Ali, Gretchen M Ehrenkaufer, Jason A Hackney, Upinder Singh

**Affiliations:** 1 Department of Microbiology and Immunology, Stanford University School of Medicine, Stanford, California, 94305-5107, USA; 2 Department of Internal Medicine and Division of Infectious Diseases, Stanford University School of Medicine, Stanford, California, 94305-5107, USA; 3 Department of Medicine, Division of Infectious Diseases, S-143 Grant Building, 300 Pasteur Drive, Stanford, CA, 94305-5107, USA

## Abstract

**Background:**

In higher eukaryotes DNA methylation regulates important biological functions including silencing of gene expression and protection from adverse effects of retrotransposons. In the protozoan parasite *Entamoeba histolytica*, a DNA methyltransferase has been identified and treatment with 5-azacytidine (5-AzaC), a potent inhibitor of DNA methyltransferase, has been reported to attenuate parasite virulence. However, the overall extent of DNA methylation and its subsequent effects on global gene expression in this parasite are currently unknown.

**Results:**

In order to identify the genome-wide effects of DNA methylation in *E. histolytica*, we used a short oligonucleotide microarray representing 9,435 genes (~95% of all annotated amebic genes) and compared the expression profile of *E. histolytica *HM-1:IMSS parasites with those treated with 23 μM 5-AzaC for up to one week. Overall, 2.1% of genes tested were transcriptionally modulated under these conditions. 68 genes were upregulated and 131 genes down regulated (2-fold change; p-value < 0.05). Sodium-bisulfite treatment and sequencing of genes indicated that there were at least two subsets of genes with genomic DNA methylation in *E. histolytica*: (i) genes that were endogenously silenced by genomic DNA methylation and for which 5-AzaC treatment induced transcriptional de-repression, and (ii) genes that have genomic DNA methylation, but which were not endogenously silenced by the methylation. We identified among the genes down regulated by 5-AzaC treatment a cysteine proteinase (2.m00545) and lysozyme (52.m00148) both of which have known roles in amebic pathogenesis. Decreased expression of these genes in the 5-AzaC treated *E. histolytica *may account in part for the parasites reduced cytolytic abilities.

**Conclusion:**

This work represents the first genome-wide analysis of DNA-methylation in *Entamoeba histolytica *and indicates that DNA methylation has relatively limited effects on gene expression in this parasite.

## Background

Methylation is an enzymatic modification of DNA that occurs post replication and epigenetically contributes to transcriptional regulation. Many important biological processes are modulated by DNA methylation including regulation of gene expression [1], development [2], and control of retrotransposon elements [3]. DNA methylation is also responsible for maintenance of chromatin structure [4] and inactivation of chromosome X in female mammals [5]. DNA methyltransferases (DNMTs) catalyze DNA methylation, which in humans and higher eukaryotes occurs predominantly at the C5-cytosine (m5C) in CpG dinucleotides [6]. DNA methylation can silence genes by directly blocking the interaction of transcription factors to their regulatory sequences [7,8]. DNA methylation can also attract methyl-binding protein, which recruits histone deacetylases and histone methyltransferases, resulting in an inactive chromatin structure [9,10]. Defects in establishing or maintaining DNA methylation patterns are associated with a number of human diseases and conditions such as cancers [11], schizophrenia [12], and aging [13].

*Entamoeba histolytica *is a protozoan parasite and the causative agent of amebic dysentery and amebic liver abscesses. These diseases result in significant morbidity and mortality worldwide, especially in developing countries, where an estimated 50 million cases of invasive amebiasis result in up to 100,000 deaths annually [14]. An active DNA methyltransferase (*Ehmeth*) of the DNA methyltransferase 2 (DNMT2) family has been characterized in *E. histolytica *[15] and ribosomal DNA [15] and a heat shock protein 100 (EHsp100) gene [16] have been shown to be methylated in this parasite. Additionally, an amebic protein which binds preferentially to methylated DNA has recently been identified (*E. histolytica *methylated LINE binding protein, *EhMLBP*) [17]. Importantly *E. histolytica *strain HM-1:IMSS grown with 5-azacytidine (5-AzaC), a potent inhibitor of DNA methyltransferase, had been shown to have significantly reduced virulence *in vitro *and *in vivo *[15]. Furthermore, the decrease in parasite virulence was reversible upon removal of the drug, indicating that drug exposure likely did not cause significant permanent mutations in the *E. histolytica *genome sequence [15]. On the other hand, over expression of *Ehmeth *in *E. histolytica *resulted in accumulation of multinucleated cells, up regulation of heat shock protein 70 (HSP70) expression, and resistance to oxidative stress [18]. All these findings suggest that DNA methylation has important biological functions in this parasite.

Information on the effects of DNA methylation in simple eukaryotes is relatively limited. 5-AzaC treatment of *Trypanosoma cruzi *epimastigotes in culture induces active cell proliferation as evident from an increase in the cell number and [3H-methyl] thymidine incorporation into DNA [19]. DNA methylation increases during development of *Dictyostelium discoideum *and DNA methyltransferase mutant cells exhibit morphological defects in late development, indicating that DNA methylation has a regulatory role in *Dictyostelium *development [2]. In ciliates, cytosine methylation occurs in transposon-like elements in the course of macronuclear differentiation in *Stylonychia lemnae *[20] and 5-AzaC treatment induces encystment in *Colpoda inflata *[21].

Several microarray-based studies have demonstrated that inhibition of promoter methylation by a drug that inhibits DNA methyltransferase results in altered gene expression [22-25]. In *Arabidopsis thaliana *transcriptional profiling revealed that inhibition of DNA methylation by 5-Aza-2-deoxycytidine (5Aza-dC) altered the expression of 1.6% of genes tested, with 73 being up-regulated and 52 down-regulated by more than 3 fold [26]. Similarly 5-Aza-dC treatment of a human gastric cancer cell line caused up-regulation of about 1.5% of the genes by at least 16-fold [22]. Treatment of esophageal squamous carcinoma cells with 5 μM of 5-Aza-dC caused at least 3-fold or more up regulation of 1.92% of 12,599 genes [24].

In order to identify the genome-wide effects of DNA methylation on gene expression in *E. histolytica*, we performed transcriptional profiling of parasites treated with and without 5-AzaC. Using a short oligonucleotide microarray representing 9,435 of the predicted 9,938 open reading frames from *E. histolytica *[27] we identified 199 genes (2.1%) as being modulated at least 2-fold by 5-AzaC. Of these, 68 genes were up-regulated and 131 genes down regulated. These data indicate that epigenetic gene silencing is operational in *E. histolytica *although it does not effect a large portion of the amebic genome.

## Results

### Treatment with 5-AzaC did not significantly affect parasite growth but reduced virulence *in vitro*

**Figure 1:**
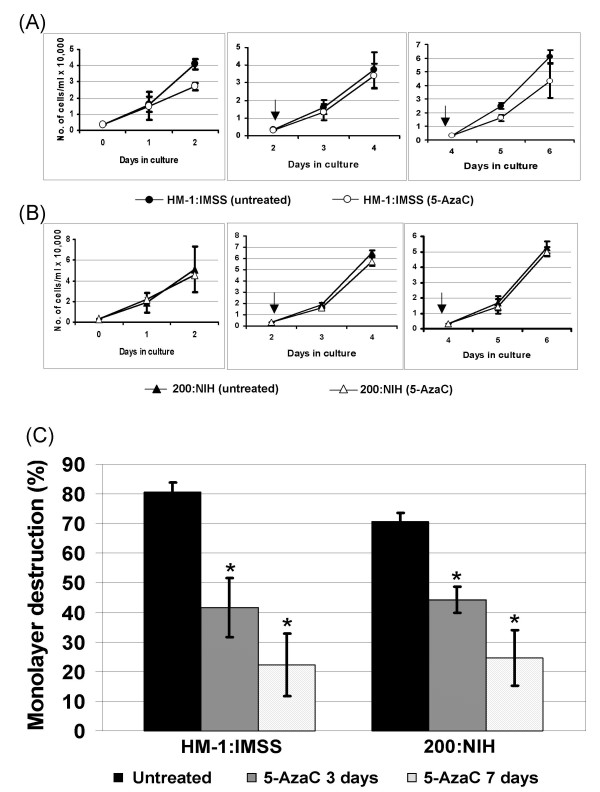
**Effects of 5-AzaC on *E. histolytica *growth and *in vitro *virulence**. **(A) **and **(B) **Growth curves for untreated and 5-AzaC treated *E. histolytica *HM-1:IMSS and *E. histolytica *200:NIH strains respectively. On day zero 50,000 trophozoites were inoculated in 15 ml culture tubes and grown with or without 23 μM 5-AzaC. On days two and four (marked by a downward arrow), 50,000 trophozoites were subcultured into fresh media and 5-AzaC added to the appropriate tubes. **(C) **Monolayer destruction of CHO cells by untreated, three-day, and seven-day 5-AzaC treated trophozoites of *E. histolytica *strains HM-1:IMSS and 200:NIH. In both parasite strains the 3-day and 7-day 5-AzaC treated parasites showed significantly decreased virulence compared to that with corresponding untreated parasites (p-value < 0.05) (shown by *).

In order to elucidate the effects of inhibiting genomic DNA methylation in *E. histolytica*, we grew parasites in 23 μM 5-AzaC for up to seven days with routine passaging and assessed growth rates and *in vitro *virulence. We used two different *E. histolytica *strains HM-1:IMSS and 200:NIH, both of which are considered virulent [28,29]. For *E. histolytica *HM-1:IMSS parasites exposed to 23 μM 5-AzaC there was some reduced growth at day 2, compared to the untreated control, however, the parasite numbers in subsequent days were consistently equivalent (Figure 1A). In *E. histolytica *200:NIH treated with 23 μM 5-AzaC, we did not observe any statistically significant effect on growth for up to seven days compared to the untreated control (Figure 1B). Additionally, we grew *E. histolytica *HM-1:IMSS for five consecutive days without routine subculturing, and did not observe any growth differences in parasites grown with or without 23 μM 5-AzaC (see Additional file 1A). We could not reliably grow parasites for >5 days without subculturing due to overcrowding of the cultures and parasite death. For the 200:NIH strain, the parasite numbers in 5-AzaC treated and untreated cultures were also equivalent at all time points tested, up to day 5 (data not shown). Under these conditions, we assessed the protein content per cell of *E. histolytica *HM-1:IMSS parasites at days 2, 3, 4, and 5 of growth, and found that there were no significant differences in protein content per cell between drug treated and untreated parasites or between parasites at different time points (see Additional file 1B). Similar data were observed for the 200:NIH strain (data not shown). We also grew the *E. histolytica *HM-1:IMSS strain (± 23 μM 5-AzaC) with routine subculturing every two days with an equal volume of culture medium and parasites passed at each time. Under these conditions, the growth of the parasites in 23 μM 5-AzaC was slightly reduced at day 2, however the rate of growth was subsequently equivalent to the untreated control at days 4 and 6 (see Additional file 1C). Overall, these data indicate that there is not a substantial and sustained effect on the growth of the *E. histolytica *parasites for up to 6–7 days in the presence of 23 μM 5-AzaC.

For both *E. histolytica *HM-1:IMSS and *E. histolytica *200:NIH, 5-AzaC exposure for 3 or 7 days resulted in parasites that were reduced in virulence (52% or 63% of untreated levels respectively at day 3 and 28% and 35% of untreated levels respectively at day 7) (Figure 1C). Exposure to 5-AzaC at 23 μM did not affect CHO cell viability and addition of 23 μM 5-AzaC to the parasites at the time of the CHO cell killing assay did not affect parasite virulence (data not shown). Thus the reduction in ability of the parasites to kill CHO cells was dependent on prior exposure of the parasites to 5-AzaC. Although this was an *in vitro *cell killing assay and thus an indirect measure of parasite virulence, our results correlate with previously published data of *in vitro *and *in vivo *virulence attenuation in *E. histolytica *HM-1:IMSS grown with 23 μM of 5-AzaC for 1 week [15,16]. Since *E. histolytica *parasite strains are genomically diverse (as shown by comparative genomic hybridizations) [30] and have genome-wide expression differences [31], it was not clear if the effects of 5-AzaC could be generalized to other *E. histolytica *strains. We have now shown that two genetically distinct, but virulent, strains of *E. histolytica*, are similarly attenuated in their virulence potential by 5-AzaC treatment. Whether this virulence attenuation is due to a direct effect of the 5-AzaC on *E. histolytica *genomic DNA methylation, is due to some other effect on the parasite, or is simply an associative phenomenon, cannot be definitively stated at present.

### Microarray expression profiling reveals genes modulated by 5-AzaC treatment

In order to identify genes whose expression was modulated by 5-AzaC treatment, expression profiling of 9,435 amebic genes was performed for untreated and 5-AzaC treated *E. histolytica *HM-1:IMSS. We chose to perform expression profiling with the *E. histolytica *HM-1:IMSS strain for a number of reasons: (i) this is the strain for which the genome sequence is available and the array was designed, (ii) transcriptome data are available for this strain from an invasive mouse model of colitis and can be used for comparative purposes [27], (iii) this is the strain for which there are published data for potential roles of the retrotransposon elements (long interspersed nuclear elements (LINEs) and short interspersed nuclear elements (SINEs)) in amebic pathogenesis and gene silencing [31,32], (iv) there are substantial genomic differences in coding and non-coding regions of the 200:NIH strain compared to HM-1:IMSS [30], and (v) the vast majority of relevant literature is for the HM-1:IMSS strain.

To identify genes affected by genomic DNA methylation, we chose to look at both early (3 days) and late (7 days) time points after 5-AzaC exposure. We chose the early time point to identify genes that are likely modulated by 5-AzaC as a primary effect (rather than a secondary down-stream effect of expression changes in other genes). However, in case certain genomic regions were more resistant to the effects of 5-AzaC, we also analyzed the parasite transcriptome after seven days of drug exposure. Importantly, for both these time points, there were no significant effects on parasite growth rates and both time points gave consistently attenuated virulence phenotypes in both *E. histolytica *strains (Figure 1 and see Additional file 1).

**Figure 2:**
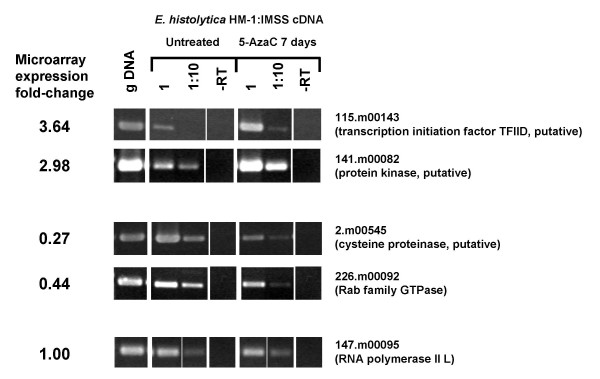
**Verification of array data by RT-PCR**. Array data were confirmed for a subset of genes by semi-quantitative RT-PCR. Total RNA was isolated from untreated and 5-AzaC treated parasites (7 days) and subjected to RT-PCR. Sequential 1:10 dilutions of cDNA were used as template for the PCR and a genomic DNA and minus RT control (-RT) were included. The microarray expression fold-change for each gene is shown based on average array data from 3 day and 7 day 5-AzaC treated parasites. Two genes (115.m00143 and 141.m00082) that were predicted to be upregulated (based on array data) after 5-AzaC exposure were confirmed by RT-PCR. Two genes (2.m00545 and 226.m00092) that were predicted to be down-regulated (based on array data) after 5-AzaC exposure were confirmed by RT-PCR. A gene whose expression did not change (based on array data) with 5-AzaC exposure (147.m00095) was found to be unchanged in the two conditions by RT-PCR. Primers used are given in Table 1.

Genes that are endogenously silenced by genomic DNA methylation should be transcriptionally upregulated under 5-AzaC treatment. We considered genes transcriptionally regulated if they were modulated at both time points of 5-AzaC treatment compared to the untreated controls (overall 2-fold change and p-value < 0.05). To confirm the array data, a subset of genes whose expression was modulated by 5-AzaC exposure were tested by semi-quantitative RT-PCR and in all cases the array data were confirmed (Figure 2; Table 1). A total of 199 transcripts (2.1% of all amebic genes tested) were significantly modulated with 68 genes upregulated and 131 genes down regulated in 5-AzaC treated parasites compared to control, untreated parasites (Tables 2, 3, and see Additional File 4). Approximately 58% of these genes were annotated as hypothetical or of unknown function. Whether all the genes that were upregulated by 5-AzaC treatment are methylated or were secondarily controlled by other genes cannot be definitively stated. However, we required that genes be transcriptionally modulated at both early and late time points, thus hopefully minimizing the potential secondary downstream effects. Genes whose expression decreases with 5-AzaC treatment are expected to be secondary or downstream effects of regulation of another gene. Inhibition of genomic DNA methylation would not, to the best of our knowledge, be directly expected to repress gene expression [33].

Genomic regions previously known to be methylated in *E. histolytica *are the heat shock protein 100 (EHsp100), ribosomal RNA (rRNA), and heat shock protein 70 (HSP70), and the repetitive region EhMRS2 [16,18,34]. Of these, the only gene known to be transcriptionally modulated by DNA methylation is EHsp100 [16]. In the study by Bernes *et al*., the EHsp100 gene transcript could not be detected in untreated *E. histolytica *HM-1:IMSS parasites but was upregulated in 5-AzaC-treated and heat-shocked parasites [16]. This gene is represented by locus 192.m00086 in the *E. histolytica *TIGR Genome Database [35] and represented by the 64.m00187_s_at probe set in our microarray. However, in our studies this probe set showed significant hybridization under standard culture conditions in *E. histolytica *HM-1:IMSS parasites and was not modulated by inhibition of DNA methylation (Table 4; see Additional file 4). This probe set was also showed significant hybridization in the 200:NIH strain (data not shown). Since this probe set also represented a number of other genes (111.m00116, 181.m00064, 365.m00018, 482.m00014, 493.m00033, 511.m00026, 82.m00144, and 872.m00009), we confirmed the array results by RT-PCR using gene specific primers as designed by Bernes *et al *[16]. The RT-PCR analysis, specific to the EHsp100 gene, confirmed our microarray data and indicated that in our *E. histolytica *HM-1:IMSS strain the EHsp100 gene was expressed under baseline culture conditions and was not modulated by 5-AzaC treatment (see Additional file 2). Other genes associated with the heat shock or stress response were not expressed in our parasites under standard culture conditions, indicating that the parasite cultures were not generally stressed (see Additional file 4). The difference in basal EHsp100 gene expression between our cultures and those of Bernes, *et al*. [16] may be due to differences in the parasite strains. It has been shown that different clones of *E. histolytica *HM-1:IMSS have different expression levels for certain genes, including retrotransposons [31], and have other biologically distinct properties relating to gene silencing [36].

Using bisulfite treatment followed by sequencing of the coding and promoter regions we confirmed that the EHsp100 gene in our parasite strain was methylated (Table 4). However, the extent of DNA methylation in the promoter and coding regions of the EHsp100 gene in our HM-1:IMSS parasite clone was slightly lower compared to that of Bernes, *et al*. [16]. For example, position 45 of the promoter (according to the nucleotide positions referred to by Bernes, *et al*., [16]) was found to be unmethylated in 6 out of 6 clones tested in our HM-1:IMSS parasite strain while 2 out of 5 clones in Bernes' HM-1:IMSS showed evidence of methylation. Similarly, for other positions fewer clones in our strain showed methylation compared to their parasites (data not shown). Whether this slight difference in DNA methylation in our strain compared to that of Bernes, *et al*. [16] was responsible for the baseline higher expression under standard conditions is not clear at present. While our microarray does not contain probe sets for rRNA genes, other genes previously known to contain cytosine methylation such as HSP70 (218.m00068) were not modulated by 5-AzaC treatment. In fact, it has been shown that the expression of HSP70 is not regulated by its cytosine methylation [18]; thus, our data are consistent with this observation. We also identified other genes, whose transcription was marginally modulated (but which did not meet our 2-fold and p-value < 0.05 cutoff) but whose promoters were methylated (687.m00016, 3.m00674, 160.m00098, 26.m00304) (Table 4).

Thus, there appear to be at least two subsets of genes in *E. histolytica*. First are those that are endogenously silenced by genomic DNA methylation and for which 5-AzaC treatment induces significant transcriptional de-repression (such as 141.m00082, 115.m00143, and 97.m00140). The second category of genes are those that have genomic DNA methylation, but are not endogenously silenced (such as EHsp100, HSP70, 687.m00016, 3.m00674, 160.m00098, 26.m00304) [18]. Whether the second category of genes (except for EHsp100 which is sensitive to 5-AzaC treatment) are more resistant to 5-AzaC [37] or are those in which the methylation profile is not due to methyltransferases but instead due to other factors, such as dsRNA-directed methylation [38], is not clear at present.

### RT-PCR confirmed the array data

In order to confirm the microarray data, we isolated RNA from 5-AzaC treated and untreated *E. histolytica *HM-1:IMSS, and performed semi-quantitative RT-PCR on five genes: two each from significantly up (115.m00143 and 141.m00082) and down-regulated gene sets (2.m00545 and 226.m00092), respectively, and one invariant gene (147.m00095), based on Affymetrix analysis. RT-PCR agreed with the microarray data for all five transcripts tested (Figure 2).

### Genomic DNA methylation apparently silences a small subset of amebic genes

Of the total 199 genes modulated in 5-AzaC treated *E. histolytica *HM-1:IMSS, 68 (0.7% of all amebic genes) were significantly up regulated by inhibition of genomic DNA methylation (Table 2). Approximately 72% of these were annotated as hypothetical proteins. Among the others were four putative protein kinases (401.m00029, 194.m00103, 141.m00082, and 159.m00103), two BspA-like leucine rich repeat protein genes (371.m00031 and 64.m00173), transcription initiation factor TFIID (115.m00143), and a DNA mismatch repair protein gene (93.m00158). There are two other putative transcription initiation factor TFIIDs annotated in the *E. histolytica *genome (324.m00040 and 40.m00208), both of which were expressed in detectable levels in untreated HM-1:IMSS parasites and were not regulated by 5-AzaC treatment (see Additional file 4). It is not clear whether these two TFIID genes contain genomic DNA methylation. In contrast, the 115.m00143 gene had very low basal gene expression and was up regulated in 5-AzaC treated parasites, and has genomic DNA methylation of the coding region (Table 4). To the best of our knowledge, no such basal transcription factors have been shown to be modulated by DNA methylation in other systems. In prokaryotes DNA methylation is involved in DNA mismatch repair, and it was interesting to see that the amebic DNA mismatch repair gene was also regulated by DNA methylation [39].

Overall, 53 genes went from undetectable baseline expression (microarray signal < 0.2) to a detectable expression levels after treatment by 5-AzaC; only 33 genes were up regulated by ≥ 3-fold, and only 4 up-regulated by ≥ 10-fold (Table 2). Thus, cytosine methylation apparently silences a relatively small number of amebic genes. As we have shown that 23 μM 5-AzaC treatment for seven days was adequate for inhibiting methylation of the EHsp100 gene (Table 4), we disfavor a technical reason for this observation. However, we cannot exclude that this parasite contains other types of rare DNA methylation (adenine methylation or N-4 cytosine methylation) [40-42], which remained unaffected by 5-AzaC treatment or that some amebic genomic regions have 5-methylcytosine that is resistant to 23 μM of 5AzaC treatment. In *Arabidopsis thaliana *treatment with 5-Aza-dC resulted in up-regulation of 0.9% of all genes in the array by ≥ 3-fold [26]. This suggests that DNA methylation silences a relatively small number of genes in these two systems.

### Identification of genomic DNA methylation by bisulfite treatment and DNA sequencing

It has been previously shown that the *E. histolytica *HM-1:IMSS EHsp100 gene contained methylation in both the promoter and coding regions [16]. In order to confirm these data for our *E. histolytica *HM-1:IMSS strain, we performed a bisulfite reaction followed by strand-specific PCR of the EHsp100 gene. This procedure converts unmethylated cytosine residues to uracil, giving rise to thymine after amplification by PCR [43]. Only methylated cytosines are refractory to the deamination and are still seen as cytosines after PCR amplification. We demonstrated that the EHsp100 gene is methylated at both the coding and promoter regions and that 5-AzaC treatment (23 μM for 7 days) significantly inhibited methylation of this gene (Table 4). The level of demethylation we observed was similar to that observed by Bernes, *et al*. [16]. This confirmed that the 5-AzaC was working as expected under our usage conditions.

In order to determine the extent of genomic DNA methylation in genes that were transcriptionally up regulated by 5-AzaC treatment, we performed sodium bisulfite treatment, PCR and sequencing of three genes significantly upregulated by 5-AzaC treatment (141.m00082, 97.m00140, and 115.m00143). These three genes had extremely low basal gene expression (0.12 ± 0.07) (median ± standard error). We identified that two genes (141.m00082 and 97.m00140) contained cytosine methylation in both coding and corresponding promoter regions, while the third one (115.m00143) showed only limited methylation in the coding region (Table 4). Cytosine methylation in the coding region has been implicated in gene silencing in other systems [44] and appears to be responsible for the endogenous silencing of the 115.m00143 gene in *E. histolytica*. Overall the trend was for greater methylation in coding than promoter regions, as also seen by Bernes, *et al*. [16]. Thus, our microarray data identified novel genes whose expression was endogenously silenced by genomic DNA methylation.

Since the number of genes that were apparently regulated by DNA methylation was limited, we investigated whether genes that were somewhat transcriptionally modulated (but that did not meet our significance fold-change or p-value criteria) may also be genomically methylated. Four such genes were subjected to bisulfite treatment and sequencing: 687.m00016, 3.m00674, 160.m00098, and 26.m00304. We picked genes that were transcriptionally upregulated early (day 3 of 5-AzaC treatment) (see Additional file 4) in order to improve our chances of finding genes partially regulated by methylation. Surprisingly, all four genes had substantial methylation in their promoter regions (Table 4). Interestingly, despite the significant extent of genomic DNA methylation, these four genes were not transcriptionally silenced under basal conditions (basal expression levels, 6.04 ± 3.25) (median ± standard error). A similar trend was previously noticed for the HSP70 gene, which has genomic methylation, but is not transcriptionally modulated by DNA methylation [18]. Additionally, in our strain of *E. histolytica *HM-1:IMSS, the EHsp100 gene is methylated but not endogenously silenced or transcriptionally modulated by 5-AzaC treatment (Table 4).

Thus, the extent of genomic DNA methylation in *E. histolytica *is likely greater than that indicated by the expression profiling as not all genomic regions that are methylated appear to be either transcriptionally silenced or substantially regulated by 5-AzaC treatment. We do not feel that this represents a technical flaw (e.g. the amount of 5-AzaC used or duration of drug exposure) as 23 μM 5-AzaC for 7 days did completely abolish methylation at the Hsp100 coding region (Table 4). Additionally, higher does of 5-AzaC (50 μM and higher) are rapidly lethal to the parasite (our observations and [15]). As mentioned earlier, each gene that is methylated in *E. histolytica *does not necessarily respond to 5-AzaC treatment [18]. Similar observations have also been made in several insect systems. DNA methylation of defensin gene in the lepidopteran *Mamestra brassicae *is not involved in gene silencing as it is constitutively expressed [45]. Likewise, highly expressed genes in *Myzus persicae *[46,47] and *Planococcus citri *[48,49] contain DNA methylation.

As expected, a gene whose expression levels significantly decreased after 5-AzaC treatment showed no cytosine methylation (226.m00092). Genes whose expression decreases after exposure to 5-AzaC would not be expected to be directly transcriptionally modulated by drug treatment, but likely change expression levels as a downstream effect of other transcriptional changes in the parasites.

### The majority of genomic DNA methylation in *E. histolytica *is not at CG di-nucleotides

In most systems, genomic DNA methylation occurs at CpG dinucleotides [50]. The skewed AT content of the amebic genome (~75% A/T) predicts that methylation will frequently not occur at CpG dinucleotides [51]. The majority of methylations we identified were located in non-CpG sites, confirming previous data for the EHsp100 gene [16]. Non-CpG methylation was also detected in *Entamoeba invadens *[52]. In order to determine if we could bioinformatically predict the location of potential DNA methylation in *E. histolytica *genomic DNA, we analyzed the frequencies of mono- and di-nucleotides in genes transcriptionally regulated by 5-AzaC compared to the whole genome. No significant differences in any of the mono or di-nucleotides were identified in up-regulated or down-regulated genes compared to the whole genome (Table 5). Thus, in the *E. histolytica *genome the frequency of the mono- or di-nucleotide occurrence was in itself not predictive of transcriptional regulation by genomic DNA methylation.

### Genes adjacent to retrotransposable elements are not substantially silenced by genomic DNA methylation

Retrotransposable elements can have substantial effects on genome structure and gene expression [53]. One method by which they can modulate gene expression is by regulating the expression of adjacent genes by methylation [54]. Retrotransposable elements are abundant in *E. histolytica *and some are maintained in hundreds of copies per genome [55]. Previously we have identified that two such retrotransposable elements, LINEs and SINEs, have higher expression in virulent strains of *E. histolytica *HM-1:IMSS compared with nonvirulent *Entamoeba *[31]. In the reptile parasite *Entamoeba invadens *the reverse transcriptase of LINE (RT LINE) contains DNA methylation and it has been suggested that both *E. invadens *and *E. histolytica *possess a mechanism for DNA methylation of LINE retrotransposons [52]. It is also not clear how many fully functional LINE and SINE elements are present in *E. histolytica *(the majority of those that are sequenced are mutated/truncated) but we do know that at least some of the LINE and SINE elements are transcribed [31].

We were unable to detect the transcriptional profile of LINEs/SINEs as there are no probe sets representing these regions in our microarray. We wished to determine if genes adjacent to LINEs or SINEs were preferentially modulated by 5-AzaC treatment. The normalized expression values for all genes adjacent to LINEs or SINEs were 0.761 ± 0.09 (median ± std error) and 0.509 ± 0.362 (median ± std error), respectively, in untreated *E. histolytica *HM-1:IMSS, and 0.633 ± 0.207 (median ± std error) and 0.449 ± 0.786 (median ± std error), respectively, in 5-AzaC treated *E. histolytica *HM-1:IMSS. Thus, we did not identify any significant association of genes that were modulated by 5-AzaC and their genomic proximity to LINE or SINE elements compared with untreated parasites. This finding indicates that even if the LINE/SINE elements in *E. histolytica *contain DNA methylation they do not necessarily co-regulate adjacent genes by this mechanism.

Additionally, we identified that adjacent loci in the same scaffold did not show co-regulation by DNA methylation (data not shown). This phenomenon is also observed in *Arabidopsis thaliana*, in which transcriptional profiling reveals that 5-Aza-dC responsive genes are distributed randomly throughout the chromosome arms and are not clustered in any obvious pattern [26].

### Effect of 5-AzaC on *E. histolytica *virulence

Virulent strains of *E. histolytica *HM-1:IMSS and 200:NIH grown with 23 μM of 5-AzaC for three or seven days showed no substantial growth differences but displayed severely reduced virulence phenotype compared to untreated parasites (Figure 1) [15]. Importantly, others have demonstrated that the effect of 5-AzaC on parasite virulence is reversible and parasite virulence returns to baseline when drug exposure is withdrawn [15]. Whether the effect of 5-AzaC on parasite virulence is simply an associative or causal finding is unclear at present. However, we did find some genes that were down regulated in 5-AzaC treated parasites, which have previously been shown to function as virulence determinants (Table 3). These included a putative cysteine proteinase (2.m00545), lysozyme (52.m00148), and a myosin Ca-binding protein (1.m00663). Cysteine proteinases (CP) are important virulence determinants in *E. histolytica *and serve a number of functions including degradation of colonic mucin and extracellular matrix [56]. Genetic proof of their importance in virulence has been shown as parasites in which members of the CP family are down regulated are less virulent *in vitro *and *in vivo *[57,58]. Lysozymes are important for degrading intracellular bacteria and thus play an important role in *E. histolytica *virulence [59]. Motility is an important aspect of amebic pathogenesis and parasites that are altered in their myosin II are less virulent [60]. Likewise, *Toxoplasma gondii *myosin A has been implicated in powering parasite gliding and host cell invasion [61]. In addition, five protein kinase genes and eight GTPase family protein genes were also down regulated significantly in 5-AzaC treated parasites. Protein kinases can affect the functions of a large number of genes and the GTPase gene family is important in vesicle trafficking, an important aspect of biology in the professionally phagocytic *E. histolytica *[62]. Thus, some roles of these genes in amebic pathogenesis can be anticipated, although functional genetic proof will be necessary to assign a definitive role to these genes in amebic virulence. Importantly, eight of the genes down regulated under 5-AzaC treatment also had decreased expression in a non-virulent strain of *E. histolytica*, Rahman, compared to the virulent *E. histolytica *HM-1:IMSS (108.m00122, 136.m00107, 22.m00298, 297.m00061, 32.m00239, 37.m00215, 442.m00023, and 460.m00024) (Table 3) [31,63]. Interestingly, in *E. histolytica *Rahman, treatment with 23 μM 5-AzaC did not significantly change the transcriptional profile of these genes (data not shown). A number of genes that have decreased expression in avirulent *Entamoeba *strains and species have been shown to be virulence determinants including the Gal/GalNAc light subunit lectin [64], *CP*1 and *CP*5 [56-58], and amebapore [32]. In addition, four genes down regulated by 5-AzaC treatment (15.m00302, 2.m00545, 67.m00091, and 71.m00153) are significantly up regulated during parasite invasion in a mouse model of amebic colitis (Table 3) [27]. Since *E. histolytica *trophozoites passed through a mouse model become more virulent, some of the upregulated genes from the *in vivo *mouse model of colitis may have direct roles in virulence. Thus, data from a number of different angles indicates that genes that are down regulated by 5-AzaC treatment are good candidates on which to perform directed functional studies to elucidate their potential contributions to amebic virulence.

## Discussion

Cytosine methylation is the most frequent endogenous modification of DNA in eukaryotes and is involved in regulating gene expression [1]. DNA methylation has been implicated as having a multitude of effects in *Entamoeba histolytica*, however, the extent of DNA methylation and its overall roles in epigenetic gene silencing in this parasite were unknown. Here we report for the first time whole genome transcriptional profiling of *E. histolytica *parasites treated with 5-AzaC, an inhibitor of DNA methyltransferase. Our findings indicate that 68 genes (about 0.7% of tested genes) are endogenously silenced by methylation in this parasite.

We identified DNA methylation in the coding regions of some *E. histolytica *genes and in some instances higher levels of cytosine methylation in the coding regions compared to the promoter regions. In the transcription initiation factor TFIID gene (115.m00143) we only identified methylation in the coding region and yet this gene was transcriptionally silenced by endogenous methylation. This is consistent with the observation that DNA methylation within the body of genes may also have a dampening effect on transcriptional elongation. It has been suggested that methyl binding proteins might be involved in inhibiting elongation, either directly or by their effects on the surrounding chromatin structure [44]. A recent report of DNMT2 protein having a novel tRNA methyltransferase activity in mouse, *Arabidopsis*, and *Drosophila *suggests that the biological role of this protein is broader than was previously anticipated [65]. Interestingly, the cytoplasmic localization of the DNMT2 protein in mammalian cells is in contrast to the nuclear localization seen in *E. histolytica *[15]. It is not currently known whether the *E. histolytica *DNMT2 may also have a tRNA methyltransferase activity.

The *E. histolytica *genome is littered with retrotransposon elements of the LINE and SINE category. As a measure of genome defense, organisms often methylate these elements (and adjacent genes) in an attempt to silence them and prevent adverse effects of random transposition (reviewed in [3]). Our analysis indicates that in *E. histolytica *HM-1:IMSS genomic regions adjacent to the LINE and SINE elements are not significantly modulated by treatment with 5-AzaC. However, methylation of some LINE elements in *Entamoeba *have been reported and a DNA methylation based mechanism for controlling these elements is anticipated [52]. Thus, either the methylation is specific to the LINE elements (which we were not able to address in our analysis as the arrays do not contain probes for these regions) and does not spread to adjacent genomic regions, or the adjacent loci have a methylation pattern that is resistant to 23 μM 5-AzaC treatment. Additionally, although a LINE element was identified as being methylated (initially isolated using affinity chromatography and an anti-5-methyl cytosine antibody) attempts to identify a specific genomic copy of the LINE element that is methylated in *E. histolytica *have been unsuccessful to date [52]. Taken together the data indicate that methylation is involved to some degree in controlling LINEs in *E. histolytica*, but is likely not a genome-wide phenomenon and that other mechanisms to control retrotransposition in *E. histolytica *likely exist.

Despite the relatively few changes in gene expression induced by 5-AzaC treatment (68 genes upregulated and 131 genes down-regulated), there was marked reduction in monolayer destruction by two virulent strains of *E. histolytica *(HM-1:IMSS and 200:NIH). Since the two parasite strains are genetically distinct [30] and have unique expression profiles [63], the consistent effects of 5-AzaC treatment on amebic phenotype are interesting. Thus, genes that had differential expression in 5-AzaC treated parasites (especially those whose expression decreased by 5-AzaC treatment) deserve further directed functional studies to characterize their potential roles in parasite virulence. Importantly, it has previously been reported that the decrease in parasite virulence by exposure to 5-AzaC was reversible upon removal of the drug, suggesting that drug exposure likely did not impart significant permanent mutations in the *E. histolytica *genome [15].

Our findings indicate that, although DNA methylation is operational in *E. histolytica*, this epigenetic mechanism of gene expression regulation affects a relatively small portion of amebic genes.

## Conclusion

DNA methylation has roles in many crucial biological functions including regulation of gene expression. The genome-wide extent of DNA methylation and its subsequent effects on gene expression are unknown in many eukaryotes. We investigated the effects of DNA methylation in the protozoan parasite *Entamoeba histolytica *using whole genome expression profiling of parasites treated with 5-azacytidine, a potent inhibitor of DNA methyltransferase. Drug treatment modulated the expression of ~2.1% of all amebic genes including 68 genes that were up-regulated and 131 genes that were down regulated.

## Methods

### Parasite culture, 5-AzaC treatment, and growth curves

Trophozoites of *E. histolytica *HM-1:IMSS and *E. histolytica *200:NIH were maintained under axenic conditions in trypticase-yeast extract iron-serum medium (TYI-S-33) supplemented with 15% adult bovine serum (Sigma), at 36.5°C as previously described [66]. The identity of both strains was confirmed by strain-specific PCR and RFLP analysis [67]. Trophozoites in log phase of growth were used in all experiments. To inhibit genomic DNA methylation, *E. histolytica *trophozoites were grown with 23 μM 5-AzaC (TRC, Canada) for up to one week with routine subculturing performed every 48 hours. For the growth curves 50,000 log-phase trophozoites were inoculated in 15 ml of TYI-S-33 media in the presence or absence of 23 μM 5-AzaC, grown under standard culture conditions as outlined above, and the parasite number recorded every 24-hours. Growth curves were performed a minimum of three times. To grow parasites without subculturing, we seeded 15,000 log-phase trophozoites in 15 ml of TYI-S33 media in the presence or absence of 23 μM 5-AzaC under standard culture conditions and monitored the parasite count every day up to day 5. In order to further assess the growth kinetics of the parasites in 23 μM 5-AzaC, 50,000 log-phase trophozoites were seeded in 15 ml of TYI-S33 media in the presence or absence of 23 μM 5-AzaC and subcultured every two days with equal inoculums of media and parasites being passed each time. Cell counts were recorded at days 2, 4, and 6 after initiation of growth in 5-AzaC.

### Protein concentration determination

Protein concentration was determined using the method of Bradford [68]. Briefly, trophozoites were lysed on ice for 2 min using Cell Culture Lysis Reagent (Promega) supplemented with protease inhibitors (500 μM AEBSF, 1 μM leupeptin, 1 μM E-64d). Cell membranes were pelleted by centrifugation for 30 sec at 14,000 × g. A standard curve for the Bradford assay was determined using bovine serum albumin as a reference. Protein concentration of the cell lysate was determined relative to this standard curve. The protein concentration (ng/cell) was determined for each time point of interest.

### Chinese Hamster Ovary (CHO) cell culture and *in vitro *CHO cell monolayer destruction assay

CHO cells were maintained in Dulbecco's minimal essential media (DMEM) supplemented with 10% fetal bovine serum, penicillin (100 U/ml), and streptomycin (100 μg/ml). CHO cell monolayer destruction by *E. histolytica *trophozoites was performed according to Bracha and Mirelman [69] with some modifications. Briefly 200,000 trophozoites of *E. histolytica *strain HM-1:IMSS or 200:NIH grown with or without 23 μM of 5-AzaC for three or seven days were incubated with confluent CHO cell monolayers in 24-well plates in 3 mls of DMEM (containing no serum) at 36.5°C for 3 hours. The integrity of the CHO cell monolayer that remained attached to the 24-well plate was determined by staining with methylene blue. The level of monolayer destruction was calculated to be inversely proportional to the amount of intact cells that remained attached to the 24-well plate. CHO cells incubated with DMEM or DMEM with 23 μM 5-AzaC served as controls. All experiments were performed at least three times. Statistical analyses were performed using a two-tailed Student's t-test and p-values < 0.05 were considered statistically significant.

### Microarray expression profiling

A custom *E. histolytica *Affymetrix array (E_his-1a520285) was designed using information from the *E. histolytica *genome sequencing project [27]. The ORF probe sets represent 9,435 of the predicted 9,938 *E. histolytica *ORFs [70]. Most of the highly repetitive sequences in *E. histolytica *such as LINEs, SINEs, rRNA genes, and tRNA genes were not included in the array as specific probe sets could not be designed. There are probe sets on the Affymetrix array representing selected intergenic regions, but these were not included in the analyses described in this paper.

Total RNA was isolated from log phase trophozoites of *E. histolytica *HM-1:IMSS and the same strain grown with 23 μM 5-AzaC for three or seven days using Trizol reagent (Invitrogen). RNA was cleaned using the RNAeasy kit (QIAGEN) according to the manufacturer's instructions and 4 μg processed for hybridization to the *E. histolytica *Affymetrix array through the Stanford University Protein and Nucleic Acid Biotechnology Facility [71] using standard Affymetrix protocols. Two arrays were generated for HM-1:IMSS parasites (not treated with drug) under mid-log conditions and two arrays were generated from HM-1:IMSS parasites treated with 5-AzaC (one each from three and seven days of treatment). Each microarray was generated from RNA harvested from parasites on different days and thus represent independent biological replicates.

### Microarray data analysis

The raw signal intensities were scaled by GCOS software so that the average of all of the probe sets on an array was 500. All data for intergenic regions was removed prior to any further analysis. The remaining data were analyzed using the GeneSpring® 7 software [72]. A global normalization was performed per chip to the median excluding the probe sets flagged absent. All the normalized expression values were averaged between the two arrays from untreated parasites (control group), and between the two arrays from three day and seven day 5-AzaC-treated parasites (treated group). We assumed that the expression variances were equal for two arrays within a particular group. To eliminate genes that were not expressed under either of the two conditions, we filtered out probe sets that had an average normalized expression value of < 0.1 in both groups analyzed (control and 5-AzaC-treated) as we have previously determined that genes with that expression level are not routinely detectable by RT-PCR (our unpublished observations). In order to identify genes that were significantly modulated in presence of 5-AzaC we used two criteria: (1) expression fold change ≥ 2-fold, and (2) a p-value < 0.05.

### RNA isolation and RT-PCR

Total RNA was extracted from log phase *E. histolytica *HM-1:IMSS parasites using TRIZOL (Invitrogen) reagent according to the manufacturer's instructions. DNA contamination in the RNA preparation was removed by DNAse treatment. Reverse transcription was performed to generate cDNA for RT-PCR using SuperScript™ II Reverse Transcriptase kit (Invitrogen) according to the manufacturer's instructions. PCR was performed with serial 10-fold dilutions of cDNA; a negative control was included with each reaction. Primers used in RT-PCR are listed in Table 1.

### Sodium bisulfite treatment and PCR amplification

*E. histolytica *HM-1:IMSS genomic DNA was isolated using standard Phenol:Chloroform method, and RNA contamination was removed by treating with RNaseA (Invitrogen) [30]. Sodium bisulfite treatment of *E. histolytica *genomic DNA (~2 μg) was performed according to the method described by Clark and Warnecke [43]. For each sample, following bisulfite treatment, two PCR amplifications were performed, each consisting of 50 cycles. Primers used to amplify promoters and genomic regions following bisulfite treatment are listed in Table 1. PCR amplified fragments derived from sodium bisulfite treated DNA were resolved on a 1.2% agarose gel, appropriate fragments excised and purified using QIAEX® II Gel Extraction Kit (QIAGEN) according to the manufacturer's instructions. Purified DNA fragments were cloned into TOPO TA vector (Invitrogen), transformed into competent *Escherichia coli *(Invitrogen), plasmid DNA isolated using QIAprep Miniprep Kit (QIAGEN), and sequenced using an ABI PRISM 310 genetic analyzer (PE Applied Biosystems) (Macrogen, Korea). Six plasmid clones were sequenced for each gene fragment.

### Genome structure analysis of genes modulated by 5-AzaC treatment

In order to determine the frequency of mono and di-nucleotides in the genomic regions affected by 5-AzaC treatment we performed the following analysis. Sequences for the promoter (500 bp upstream of the predicted start codon) and coding regions of all genes modulated by 5-AzaC treatment were downloaded from The Institute for Genomic Research (TIGR) website [35]. Additionally, the sequence of the most recent assembly (12/27/05) of the genome sequence was also obtained [35]. Using acustom script written in the Python programming language by JAH (see Additional file 3) we determined the mono and di-nucleotide frequency in the promoter and coding regions of genes up-regulated by 5-AzaC treatment, down-regulated by 5-AzaC treatment, and the entire genome.

To determine if the genes modulated by 5-AzaC treatment were adjacent to the retrotransposon elements (LINEs and SINEs), all occurrences of the three families of EhLINEs and EhSINEs were identified in the *E. histolytica *genome sequence using BLAST program [73] and the consensus sequence of the LINEs and SINEs [55] with an e-value cutoff of 10^-20^. The frequency of occurrence of each LINE and SINE gene family member was similar to the results identified previously [55]. We subsequently identified all genes adjacent (within 10 Kb, both upstream and downstream) to each member of EhLINE or EhSINE, considering only the gene most proximal to a given LINE or SINE. Using the hypergeometric distribution, we determined if the genes modulated by 5-AzaC treatment were more likely to be adjacent to an EhLINE or EhSINE element than by random chance (p-value < 0.05).

## List of abbreviations

5-AzaC, 5-azacytidine; 5-Aza-dC, 5-aza-2-deoxycytidine; PCR, polymerase chain reaction; RT-PCR, reverse-transcription-PCR; LINE, long interspersed nuclear element; SINE, short interspersed nuclear element; Ehmeth, *Entamoeba histolytica *methyltransferase; DNMTs, DNA methyltransferases; MBD, Methyl binding domain; m5C, C5-cytosine; DMEM, Dulbecco's minimal essential media; CHO, Chinese Hamster Ovary; ORF, open reading frame.

## Authors' contributions

IKMA and US designed the experiments and wrote the manuscript. IKMA performed the experiments, GME assisted with microarray experiments and analysis, and JAH provided bioinformatic analyses. All authors have read and approved the final manuscript.

## Supplementary Material

Additional File 1 **(A) ***E. histolytica *HM-1:IMSS grown in 23 μM 5-AzaC for five consecutive days have similar growth kinetics to untreated parasites. *E. histolytica *HM-1:IMSS log-phase trophozoites (15,000) were seeded into 15 ml glass culture tubes, cultured under standard conditions ± 23 μM 5-AzaC, and cell counts taken at days 2, 3, 4, and 5. The average and standard deviation values for each time point are shown for untreated and 5-AzaC treated parasites. There were no statistical differences in the cell counts at any given time point in 5-AzaC treated and untreated parasites. One representative experiment is shown. **(B) **Protein content per cell was determined at days 2, 3, 4, and 5 of parasite growth ± 23 μM 5-AzaC treatment for *E. histolytica *HM-1:IMSS parasites. The average and standard deviation values for each time point are shown. There were no statistically significant differences in the protein concentration per cell at any given time point in 5-AzaC treated and untreated parasites or in parasites grown for 2, 3, 4, or 5 days. One representative experiment is shown. **(C) ***E. histolytica *HM-1:IMSS grown in 23 μM 5-AzaC for six days with routine subculturing of equal volume of media/parasites have similar growth kinetics to untreated parasites after two days in 5-AzaC. *E. histolytica *HM-1:IMSS log-phase trophozoites (50,000) were seeded into 15 ml glass culture tubes and cultured under standard conditions ± 23 μM 5-AzaC. At days 2 and 4 (marked with a down arrow) an equal volume of media containing parasites grown ± 5-AzaC was inoculated into a new tube with fresh media (± 5-AzaC) and cell counts obtained at days 2, 4, and 6. The average and standard deviation values for each time point are shown for untreated and 5-AzaC treated parasites. One representative experiment is shown. Click here for file

Additional File 4 Normalized expression values for arrays from untreated and 5-AzaC treated *E. histolytica *HM-1:IMSS parasites. All array data have been made available per MIAME guidelines. Raw array data have been deposited at the NCBI Gene Expression Omnibus website (accession number GSE6635). Click here for file

Additional File 2 Verification of array data for EHsp100 (represented on the microarray by the probe set 64.m00187_s_at) by semi-quantitative RT-PCR. Total RNA was isolated from untreated and 5-AzaC treated parasites (7 days) and subjected to RT-PCR. Sequential 1:100 dilutions of cDNA were used as template for the PCR and a genomic DNA and minus RT control (-RT) were included. The microarray expression fold-change for each gene is shown based on average array data from 3-day and 7-day 5-AzaC treated parasites. Primers specific to the EHsp100 gene (192.m00086) as determined by Bernes *et al *[16] were used for the RT-PCR reaction. A gene whose expression did not change based on array data (147.m00095) was found to be unchanged in the two conditions by RT-PCR. Click here for file

Additional File 3 This Python script determines the frequencies of mono- and dinucleotides in a FASTA sequence file. This program requires a Python interpreter installed on the computer that is being used for this analysis, which can be freely downloaded [74]. Download the text of this file exactly as written (including white space). To run the program, type "python <name of script file> <name of FASTA sequence file>". Click here for file

## Figures and Tables

**Table 1:** PCR primers used in study.

**(A) Primers used in RT-PCR**					
**Probe set**	**Primer name**	**Direction**	**AT**	**Primer sequence (5' – 3')**	**Target region**
115.m00143_at	115.m00143F	S	55°C	CCAAACGATACACACCCAGA	Coding
	115.m00143R	AS	55°C	GCAATTTGAAACTTCACATCACA	Coding
141.m00082_at	141.m00082F	S	55°C	TCATTTGGAATTGTTTATGTTGG	Coding
	141.m00082R	AS	55°C	TGCACTTCCAAAATCCGTTA	Coding
2.m00545_at	2.m00545F	S	55°C	GCTGCCATGACTAATGCTGA	Coding
	2.m00545R	AS	55°C	AGCAACAGCAACTGGTCCTT	Coding
226.m00092_at	226.m00092F	S	55°C	GCAAACATGGGATACAGCAG	Coding
	226.m00092R	AS	55°C	ACAAGGCGCTTGTTCAACTT	Coding
					
**(B) Primers used in PCR from bisulfite treated DNA**					
					
**Probe set**	**Primer name**	**Direction**	**AT**	**Primer sequence (5' – 3')**	**Target region**

64.m00187_s_at	EHsp5'bis*	S	50°C	ATGAATAAGAAAGTGTGAATAATAG	Promoter
	EHsp3'bis*	AS	50°C	AACATTAATTCCACTATTTCCTACTA	Promoter
	EHsp1005'*	S	50°C	TGAGTATTTAAAGGAACTTGAAG	Coding
	EHsp3'bis*	AS	50°C	AACATTAATTCCACTATTTCCTACTA	Coding
115.m00143_at	115.mPF	S	52°C	CTTGAAGGATAAAAAATAAGTCATATTTCTA	Promoter
	115.mPR	AS	52°C	ATAATCTGCATGGCATATATTTCCTAATT	Promoter
115.m00143_at	115.mBCF	S	48°C	ATGTTTGTATCTTATTTTCTATTTTTAATTT	Coding
	115.mCR	AS	48°C	GTTCCAGTACATACAATTTTACCT	Coding
141.m00082_at	141.mBPF	S	52°C	TATTGAACACTGCTCTAAATCCACT	Promoter
	141.mBPR	AS	52°C	GTGTAGTACTGAATAAACACTTTTCTT	Promoter
	141.mBCF	S	53°C	TTGATATAACTAATTCACCTACATTCCAA	Coding
	141.mBCR	AS	53°C	GATCCTTCTCCTATTTTCTTTTCTTCT	Coding
97.m00140_at	97.mPF	S	50°C	ATTACTCCTTCCCTCTCTTCTT	Promoter
	97.mPR	AS	50°C	CCATTRTCATTTCTCTCAACC	Promoter
194.m00103_at	194.mPF	S	50°C	GACTRCACCCAATTTTCCACCT	Promoter
	194.mPR	AS	50°C	CCATCCCAATAAATTCCTTCTT	Promoter
687.m00016_at	687.mBPF	S	53°C	CTTTCARACACTTAACAAATCTTCTTCA	Promoter
	687.mBPR	AS	53°C	ARATTCTCTCAAATACACTTCCCAC	Promoter
3.m00674_at	3.mBPF	S	49°C	GTTATTCAATTATACTTTATACCAAACA	Promoter
	3.mBPR	AS	49°C	TCATTTTARTTCTTTTTTCTTATACCACT	Promoter
160.m00098_at	160.mBPF	S	50°C	CTAATAAAATTTTCTTACACCTAACTCA	Promoter
	160.mBPR	AS	50°C	CTTCTTTATTTTTTCCATAATATTCTTTCT	Promoter
26.m00304_at	26.mBPF	S	48°C	TAAAACTARACCATTTATTCAACAATT	Promoter
	26.mBPR	AS	48°C	RRTTCTTCTTCCATAATTTATTATTA	Promoter
2.m00545_at	2.mBPF	S	50°C	TTACTTCTCGTTATTCTATTAATATAAAC	Promoter
	2.mBPR	AS	50°C	GATTATTTTTTGCCATCCATTTATCAA	Promoter
	2.mBCF	S	50°C	CTAAATTTACAAAAAAACTAATATACTCTC	Coding
	2.mBCR	AS	50°C	TAGAGCCACCATTACATCCATTA	Coding
226.m00092_at	226.mBPF	S	49°C	CTATTGATATTGAAATGCAAAAAACAAT	Promoter
	226.mBPR	AS	49°C	TGATATATCAACTTAAACAAACTTACTA	Promoter
	226.mBCF	S	50°C	GTTCTTCTAGATTATTAAAAACCATTC	Coding
	226.mBCR	AS	50°C	AACACTTCCTTTATCTATTTTATTTCC	Coding

Primers used, annealing temperatures, sequences, and their targets (gene promoters or coding regions) are listed for the (A) RT-PCR and (B) bisulfite sequencing studies. The probe set targeted, the primer name, direction of the primer, annealing temperature, primer sequence, and region of each gene targeted (coding or promoter) are shown. S refers to the sense primer; AS to the antisense primer; R represents either A or G. * indicates primers taken from Bernes, *et al*., 2005 [16].

**Table 2:** Genes up-regulated in 5-AzaC treated *E. histolytica *HM-1:IMSS parasites as compared to untreated controls.

**Probe set**	**Baseline expression level**	**Fold-change**	**p-value**	**Annotation**
*763.m00027_s_at*^*a*^	0.01	28.97	0.024	hypothetical protein
*120.m00098_x_at*	0.01	14.35	0.011	hypothetical protein
329.m00054_at	0.01	13.06	0.017	hypothetical protein
253.m00081_x_at	0.02	10.61	0.041	hypothetical protein
16.m00301_at	0.01	9.75	0.006	hypothetical protein
228.m00060_x_at	0.02	9.43	0.007	AIG1 family protein, putative (GO:0004765)
244.m00069_x_at	0.01	9.23	0.021	hypothetical protein
371.m00031_x_at	0.01	8.69	0.006	BspA-like leucine rich repeat protein, putative
295.m00030_at	0.02	8.11	0.036	conserved hypothetical protein
64.m00173_x_at	0.01	7.95	0.043	BspA-like leucine rich repeat protein, putative
94.m00138_at	0.02	7.09	0.033	hypothetical protein
36.m00220_s_at^b^	0.02	5.78	0.005	hypothetical protein
432.m00029_x_at	0.08	5.32	0.001	conserved hypothetical protein
1087.m00005_at	0.03	5.12	0.038	hypothetical protein
401.m00029_at	1.11	4.97	0.022	protein kinase, putative (GO:0005524)
92.m00177_x_at	0.05	4.39	0.009	hypothetical protein
86.m00156_s_at^c^	0.09	4.06	0.036	glutamine cyclotransferase, putative
287.m00045_x_at	0.03	3.93	0.026	hypothetical protein
450.m00030_at	0.12	3.90	0.043	hypothetical protein
2.m00587_x_at	0.04	3.83	0.009	hypothetical protein
115.m00143_at*	0.12	**3.64**	**0.001**	**transcription initiation factor TFIID, putative (GO:0003677)**
420.m00021_x_at	0.03	3.59	0.007	hypothetical protein
27.m00245_at	0.03	3.55	0.017	hypothetical protein
103.m00185_at	6.55	3.32	0.023	Fe-hydrogenase, putative (GO:0005489)
194.m00103_at*	0.07	3.27	0.029	protein kinase, putative (GO:0005524)
26.m00274_x_at	0.07	3.22	0.039	hypothetical protein
935.m00010_at	0.11	3.15	0.009	hypothetical protein
*93.m00158_s_at*^*d*^	0.10	3.10	0.014	DNA mismatch repair protein mutS, putative (GO:0006298)
97.m00140_at*	0.08	3.10	0.006	hypothetical protein
*138.m00086_x_at*	4.75	3.09	0.023	hypothetical protein
66.m00163_s_at	0.03	3.08	0.010	hypothetical protein
12.m00286_at	0.04	3.06	0.025	hypothetical protein
295.m00044_x_at	0.07	3.04	0.017	conserved hypothetical protein
141.m00082_at*	0.32	**2.98**	**0.010**	**protein kinase, putative (GO:0004672)**
267.m00069_at	0.04	2.96	0.001	hypothetical protein
4.m00648_x_at	0.04	2.89	0.032	hypothetical protein
112.m00119_at	0.04	2.85	0.035	hypothetical protein
472.m00058_s_at^e^	0.08	2.83	0.008	hypothetical protein
204.m00098_x_at	0.22	2.77	0.008	hypothetical protein
583.m00011_at	0.41	2.69	0.026	hypothetical protein
*62.m00174_x_at*	0.06	2.66	0.037	hypothetical protein
55.m00164_at	0.07	2.63	0.002	hypothetical protein
273.m00075_x_at	0.05	2.59	0.042	hypothetical protein
249.m00077_x_at	0.06	2.55	0.049	hypothetical protein
289.m00092_at	0.05	2.53	0.014	hypothetical protein
464.m00030_at	0.05	2.53	0.010	hypothetical protein
1.m00665_at	1.11	2.53	0.046	hypothetical protein
*51.m00150_x_at*	0.05	2.49	0.009	hypothetical protein
159.m00103_x_at	0.43	2.44	0.044	protein kinase, putative (GO:0004672)
19.m00288_at	0.07	2.36	0.005	hypothetical protein
308.m00054_x_at	0.05	2.34	0.009	hypothetical protein
5.m00447_x_at	0.12	2.33	0.016	hypothetical protein
51.m00167_at	0.05	2.33	0.042	hypothetical protein
27.m00266_x_at	0.06	2.30	0.050	ELL complex EAP30 subunit, putative
222.m00072_at	0.09	2.30	0.040	SPRY domain protein
183.m00091_x_at	8.10	2.30	0.026	acyl-CoA synthetase, putative (GO:0008152)
289.m00067_s_at^f^	0.09	2.30	0.004	short chain dehydrogenase family protein
43.m00197_x_at	7.97	2.24	0.024	cell adhesion protein (GO:0007155)
54.m00234_s_at^g^	1.09	2.22	0.037	hypothetical protein (GO:0005554)
*4.m00613_x_at*	0.12	2.21	0.007	hypothetical protein
48.m00224_s_at^h^	0.71	2.21	0.018	acetyltransferase, putative
75.m00156_x_at	0.11	2.17	0.007	myotubularin, putative
25.m00258_x_at	0.38	2.16	0.032	hypothetical protein
34.m00243_x_at	4.14	2.15	0.028	glucosamine 6-phosphate N-acetyltransferase, putative (GO:0008080)
314.m00052_x_at	0.07	2.12	0.041	hypothetical protein
21.m00282_at	0.21	2.08	0.038	hypothetical protein
528.m00022_at	0.10	2.03	0.018	hypothetical protein
48.m00192_at	0.17	2.01	0.013	hypothetical protein

^a^763.m00027_s_at also represents 190.m00085 (hypothetical protein), 239.m00065 (hypothetical protein), 278.m00067 (hypothetical protein), 330.m00074 (hypothetical protein), 43.m00171 (hypothetical protein) and 458.m00061 (hypothetical protein)

^b^36.m00220_s_at also represents 36.m00212 (hypothetical protein)

^c^86.m00156_s_at also represents 119.m00135 (glutamine cyclotransferase, putative)

^d^93.m00158_s_at also represents 153.m00091 (DNA mismatch repair protein mutS, putative)

^e^472.m00058_s_at also represents 361.m00052 (hypothetical protein)

^f^289.m00067_s_at also represents 180.m00106 (short chain dehydrogenase family protein)

^g^54.m00234_s_at also represents 169.m00132 (hypothetical protein) and 406.m00049 (hypothetical protein)

^h^48.m00224_s_at also represents 309.m00047 (acetyltransferase, putative)

The probe set, baseline expression value in untreated *E. histolytica *HM-1:IMSS trophozoites, fold change in 5-AzaC treated parasites, p-value, and gene annotation are shown. Genes in bold are those for which RT-PCR confirmation was performed. Genes in italics are genes whose expression is significantly higher in *E. histolytica *Rahman compared to *E. histolytica *HM-1:IMSS (p-value 0.0002). Genes with normalized expression values >0.20 can routinely be detected by RT-PCR. Genes marked by * are those for which bisulfite sequencing was performed. For annotations, GO IDs are given where available and are provided within parentheses.

**Table 3:** A subset of genes down-regulated in 5-AzaC treated *E. histolytica *HM-1:IMSS parasites as compared to untreated controls.

**Probe set**	**Baseline expression**	**Fold-change**	**p-value**	**Annotation**
**Potentially virulence associated**				
2.m00545_at*^£^	4.12	0.27	0.033	cysteine proteinase, putative (GO:0006508)
52.m00148_at	0.96	0.46	0.018	lysozyme, putative
1.m00663_at	6.21	0.36	0.006	myosin calcium-binding light chain, putative (GO:0005509)
				
**Protein kinases**				
35.m00259_at	1.76	0.48	0.013	protein kinase, putative (GO:0005524)
67.m00091_x_at^£^	3.42	0.37	0.033	protein kinase, putative (GO:0005524)
99.m00190_s_at	0.39	0.46	0.040	protein kinase, putative (GO:0006468)
466.m00033_x_at	0.51	0.47	0.003	protein kinase, putative (GO:0005524)
264.m00068_at	2.29	0.44	0.029	protein kinase, putative (GO:0005524)
				
**GTPases**				
106.m00140_at	2.39	0.48	0.025	Rap Ran GTPase activating protein, putative
2.m00606_x_at	0.20	0.36	0.005	Rab family GTPase (GO:0005524)
226.m00092_at*	0.34	0.44	0.008	Rab family GTPase
30.m00257_at	4.28	0.50	0.023	Rab family GTPase (GO:0005524)
34.m00273_x_at	3.22	0.43	0.040	Rab family GTPase (GO:0005524)
71.m00153_x_at^£^	2.21	0.47	0.002	Rho family GTPase (GO:0005525)
8.m00366_s_at^a^	1.15	0.46	0.021	Rho GTPase activating protein, putative
171.m00089_at	4.88	0.35	0.044	GTPase activating protein, putative (GO:0003924)
				
**Genes with decreased expression in *E. histolytica *(Rahman) compared to *E. histolytica *(HM-1:IMSS)**				
108.m00122_at	32.12	0.21	0.036	hypothetical protein
136.m00107_at	1.20	0.38	0.030	hypothetical protein
22.m00298_at	4.12	0.43	0.002	hypothetical protein
297.m00061_at	5.00	0.35	0.010	hypothetical protein
32.m00239_at	0.93	0.46	0.039	N-acetylglucosaminyl transferase, putative
37.m00215_at	1.90	0.46	0.035	hypothetical protein
442.m00023_x_at	0.82	0.44	0.006	hypothetical protein
460.m00024_s_at^b^	2.18	0.27	0.015	hypothetical protein
				
**Genes overlap with day-1 & day-29 significantly up-regulated genes from *in vivo *mouse model (Gilchrist *et al*, 2006)**^£^				
15.m00302_at	4.05	0.41	0.030	hypothetical protein
2.m00545_at*	4.12	0.27	0.033	cysteine proteinase, putative (GO:0006508)
67.m00091_x_at	3.42	0.37	0.033	protein kinase, putative (GO:0005524)
71.m00153_x_at	2.21	0.47	0.002	Rho family GTPase (GO:0005525)
				
**Miscellaneous**				
338.m00049_s_at^c^	4.30	0.42	0.020	LIM domain protein (GO:0008270)
10.m00349_x_at	4.30	0.44	0.024	paxillin, putative (GO:0008270)
35.m00253_at	1.74	0.26	0.036	iron-sulfur flavoprotein, putative (GO:0006118)
646.m00021_s_at	0.36	0.40	0.009	iron-sulfur flavoprotein, putative
90.m00179_at	1.31	0.46	0.044	amino acid transporter, putative (GO:0006865)
139.m00118_at	3.90	0.44	0.048	histone H3, putative (GO:0005634)

^a^8.m00366_s_at also represents 58.m00170 (Rho GTPase activating protein, putative)

^b^460.m00024_s_at also represents 353.m00048 (hypothetical protein) and 353.m00049 (hypothetical protein)

^c^338.m00049_s_at also represents 321.m00056 (LIM domain protein)

The probe set, baseline expression value in untreated *E. histolytica *HM-1:IMSS trophozoites, fold change in 5-AzaC treated parasites, p-value, and gene annotation are shown. Genes are clustered according to functional categories. Genes in bold are those for which RT-PCR confirmation was performed. Genes with normalized expression values >0.20 can routinely be detected by RT-PCR. Genes marked by * are those for which bisulfite sequencing was performed. Genes that were significantly upregulated in *E. histolytica *HM-1:IMSS trophozoites passed through mice are obtained from Gilchrist *et al *[27] and are indicated by £. For annotations, GO IDs are given where available and are provided within parentheses.

**Table 4:** Compilation of bisulfite treatment and sequencing results.

**Probe set**	**Baseline expression value**	**Fold change**	**p-value**	**Annotation**	**Promoter region**		**Coding region**	
					Total bp analyzed	# methylated cytosines/# total cytosines	Total bp analyzed	# methylated cytosines/# total cytosines

**Control genes**								
64.m00187	14.05	0.90	0.731	Hsp100	455	10/62	397	48/48
64.m00187**	14.05	0.90	0.731	Hsp100**	ND	ND	397	0/48
								
**Significantly upregulated**								
141.m00082	0.32	2.98	0.010	protein kinase, putative	308	36/36	73	13/13
115.m00143	0.12	3.64	0.001	transcription initiation factor TFIID, putative	266	0/27	254	5/40
97.m00140_at	0.08	3.10	0.006	hypothetical protein	248	59/59	110	23/23
								
**Marginally upregulated**								
687.m00016_at	12.16	18.68	0.091	hypothetical protein	328	18/18	ND	ND
3.m00674_at	0.62	2.17	0.089	hypothetical protein	143	16/16	ND	ND
160.m00098_at	11.46	1.72	0.112	hypothetical protein	244	16/16	ND	ND
26.m00304_at	0.54	1.64	0.086	hypothetical protein	115	19/19	ND	ND
								
**Significantly downregulated**								
226.m00092_at	0.343	0.34	0.008	Rab family GTPase	347	0/27	41	0/6

64.m00187_s_at also represents 111.m00116, 181.m00064, 192.m00086, 365.m00018, 482.m00014, 493.m00033, 511.m00026, 82.m00144, and 872.m00009.

The probe set, baseline expression value in *E. histolytica *HM-1:IMSS trophozoites, fold change, p-value, and gene annotation are shown. For each gene the number of base pairs analyzed, the number of methylated cytosines, the number of total cytosines analyzed, and the location (promoter or coding region) are indicated. Genomic DNA from *E. histolytica *HM-1:IMSS parasites was treated with 10 M sodium bisulfite and PCR amplified. Six independent clones were analyzed for each region of interest to determine the extent of cytosine methylation; for a particular cytosine position to be indicated as methylated 4 out of 6 clones had to be methylated. ** represents genomic DNA from *E. histolytica *HM-1:IMSS parasites treated with 23 μM 5-AzaC for 7 days. ND = not done. Primers used in PCR analysis of the Hsp100 gene were specific to 192.m00086 and were identical to those used by Bernes *et al*. [16].

**Table 5:** Relative frequencies of mono- and dinucleotides in 5-AzaC modulated genes and in the whole genome.

		**Whole genome**		**Up-regulated genes**		**Down-regulated genes**	
**Nucleotide**		Coding	Promoter	Coding	Promoter	Coding	Promoter
Mononucleotide	A	0.36	0.40	0.42	0.40	0.40	0.40
	C	0.14	0.11	0.11	0.11	0.11	0.11
	G	0.14	0.12	0.16	0.12	0.15	0.12
	T	0.36	0.36	0.31	0.37	0.34	0.36

Dinucleotide	AA	0.14	0.19	0.18	0.18	0.17	0.19
	AC	0.04	0.04	0.04	0.04	0.04	0.04
	AG	0.05	0.05	0.06	0.05	0.06	0.05
	AT	0.12	0.12	0.12	0.13	0.13	0.12
	CA	0.06	0.05	0.06	0.05	0.06	0.05
	CC	0.02	0.01	0.02	0.01	0.01	0.01
	CG	0.01	0.01	0.01	0.01	0.01	0.01
	CT	0.05	0.04	0.03	0.04	0.04	0.04
	GA	0.06	0.06	0.08	0.05	0.07	0.05
	GC	0.01	0.01	0.01	0.01	0.01	0.01
	GG	0.02	0.02	0.02	0.01	0.02	0.01
	GT	0.04	0.04	0.04	0.04	0.04	0.04
	TA	0.09	0.11	0.09	0.11	0.10	0.11
	TC	0.06	0.05	0.04	0.05	0.04	0.05
	TG	0.06	0.05	0.06	0.05	0.06	0.05
	TT	0.14	0.15	0.11	0.16	0.13	0.16

The frequency of each mono and di-nucleotide was calculated for the entire genome and for the coding and promoter regions of the genes up and down-regulated by 5-AzaC. No specific association of any given mono or di-nucleotide frequency with genes that are responsive to 5-AzaC compared to the whole genome was identified.
