# Supplementary material for: Growth of the protozoan parasite Entamoeba histolytica in 5-azacytidine has limited effects on parasite gene expression
Source: BMC Genomics. 2007 Jan 5;8:7. doi: 10.1186/1471-2164-8-7 (PMC1779778; doi:10.1186/1471-2164-8-7)
Supplement: Additional File 1 — (A) E. histolytica HM-1:IMSS grown in 23 μM 5-AzaC for five consecutive days have similar growth kinetics to untreated parasites. E. histolytica HM-1:IMSS log-phase trophozoites (15,000) were seeded into 15 ml glass culture tubes, cultured under standard conditions ± 23 μM 5-AzaC, and cell counts taken at days 2, 3, 4, and 5. The average and standard deviation values for each time point are shown for untreated and 5-AzaC treated parasites. There were no statistical differences in the cell counts at any given time point in 5-AzaC treated and untreated parasites. One representative experiment is shown. (B) Protein content per cell was determined at days 2, 3, 4, and 5 of parasite growth ± 23 μM 5-AzaC treatment for E. histolytica HM-1:IMSS parasites. The average and standard deviation values for each time point are shown. There were no statistically significant differences in the protein concentration per cell at any given time point in 5-AzaC treated and untreated parasites or in parasites grown for 2, 3, 4, or 5 days. One representative experiment is shown. (C) E. histolytica HM-1:IMSS grown in 23 μM 5-AzaC for six days with routine subculturing of equal volume of media/parasites have similar growth kinetics to untreated parasites after two days in 5-AzaC. E. histolytica HM-1:IMSS log-phase trophozoites (50,000) were seeded into 15 ml glass culture tubes and cultured under standard conditions ± 23 μM 5-AzaC. At days 2 and 4 (marked with a down arrow) an equal volume of media containing parasites grown ± 5-AzaC was inoculated into a new tube with fresh media (± 5-AzaC) and cell counts obtained at days 2, 4, and 6. The average and standard deviation values for each time point are shown for untreated and 5-AzaC treated parasites. One representative experiment is shown. [file 1471-2164-8-7-S1.doc]

**HM-1:IMSS (5-AzaC)**

**HM-1:IMSS (untreated)**

**HM-1:IMSS (5-AzaC)**

**(B)**

**(A)**

**HM-1:IMSS (untreated)**
