# Supplementary material for: Growth of the protozoan parasite Entamoeba histolytica in 5-azacytidine has limited effects on parasite gene expression
Source: BMC Genomics. 2007 Jan 5;8:7. doi: 10.1186/1471-2164-8-7 (PMC1779778; doi:10.1186/1471-2164-8-7)
Supplement: Additional File 2 — Verification of array data for EHsp100 (represented on the microarray by the probe set 64.m00187_s_at) by semi-quantitative RT-PCR. Total RNA was isolated from untreated and 5-AzaC treated parasites (7 days) and subjected to RT-PCR. Sequential 1:100 dilutions of cDNA were used as template for the PCR and a genomic DNA and minus RT control (-RT) were included. The microarray expression fold-change for each gene is shown based on average array data from 3-day and 7-day 5-AzaC treated parasites. Primers specific to the EHsp100 gene (192.m00086) as determined by Bernes et al [16] were used for the RT-PCR reaction. A gene whose expression did not change based on array data (147.m00095) was found to be unchanged in the two conditions by RT-PCR. [file 1471-2164-8-7-S2.ppt]

## Slide 1
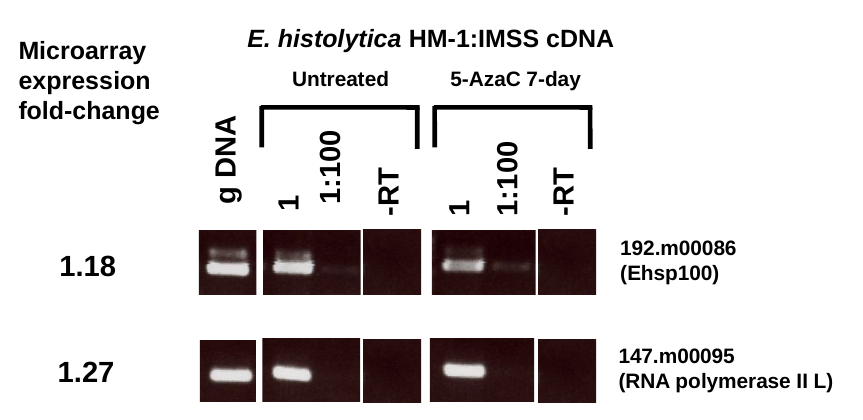

E. histolytica HM-1:IMSS cDNA
Microarray expression fold-change
Untreated
5-AzaC 7-day
g DNA
1:100
1
-RT
1
1:100
-RT
192.m00086
(Ehsp100)
1.18
1.27
147.m00095
(RNA polymerase II L)
