# Supplementary figures and images for: Growth of the protozoan parasite Entamoeba histolytica in 5-azacytidine has limited effects on parasite gene expression
Source: BMC Genomics. 2007 Jan 5;8:7. doi: 10.1186/1471-2164-8-7 (PMC1779778; doi:10.1186/1471-2164-8-7)

## Slide 1
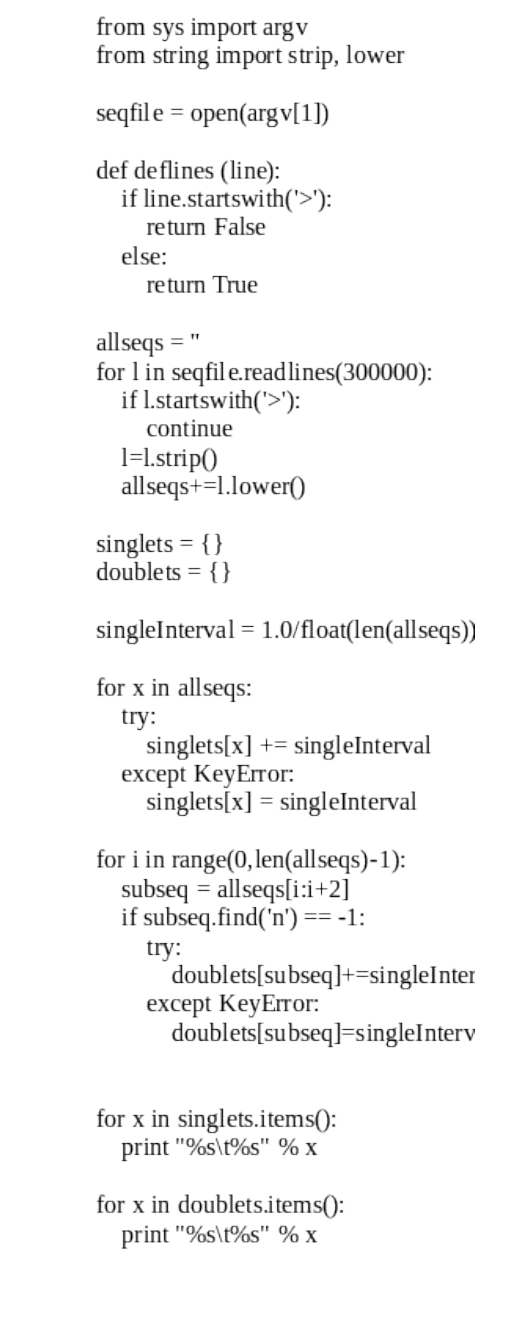

Supplement: Additional File 3 — This Python script determines the frequencies of mono- and dinucleotides in a FASTA sequence file. This program requires a Python interpreter installed on the computer that is being used for this analysis, which can be freely downloaded [74]. Download the text of this file exactly as written (including white space). To run the program, type "python ". [file 1471-2164-8-7-S3.ppt]
